# The dietary burden of phosphorus and aluminum in ready-to-eat wheat flour tortillas exceeds that of corn tortillas: Implications for patients with chronic kidney disease

**DOI:** 10.1016/j.foohum.2026.101060

**Published:** 2026-02-03

**Authors:** Kate Chander Chiang, Robert A. Yokel, Jason M. Unrine, Kamyar Kalantar-Zadeh, Ajay Gupta

**Affiliations:** a University College Dublin School of Medicine, Dublin, Ireland; b Pharmaceutical Sciences, University of Kentucky, Lexington, KY 40536-0596, USA; c Plant and Soil Sciences, University of Kentucky, Lexington, KY 40546-0091, USA; d Kentucky Water Research Institute, University of Kentucky, Lexington, KY 40506-0107, USA; e Department of Epidemiology, University of California Los Angeles, CA 90024, USA; f Division of Nephrology, Hypertension and Kidney Transplantation and Department of Medicine, University of California Irvine (UCI) School of Medicine, 92697, USA

**Keywords:** Aluminum, Chronic kidney disease, Food additives, Phosphorus, tortilla

## Abstract

**Background:**

Ready-to-eat, shelf-stable tortillas contain several phosphorus- and aluminum-containing additives that may increase the risk of adverse events in patients with chronic kidney disease (CKD).

**Objective:**

The present study aimed to measure the dietary aluminum and phosphorus burden in store-bought corn and wheat flour tortillas.

**Design:**

This cross-sectional study analyzed the elemental content of commercially available corn and wheat flour tortillas collected from 2019 to 2020.

**Setting:**

Twenty-one elements were quantified by ICP-MS and ICP-OES from 14 corn and 13 wheat flour tortilla brands purchased from local supermarkets in Southern California in 2019–2020.

**Statistical analyses performed:**

Multiple group comparisons were analyzed by one-way ANOVA with Tukey’s post hoc test. Outliers were identified using the ROUT test on GraphPad/PRISM and statistical significance was determined using nonparametric Mann-Whitney and Kruskal-Wallis tests. All conditions (soft corn, hard corn, and wheat) were compared using Dunn’s multiple comparison test.

**Results:**

Ready-to-eat wheat flour tortillas generally contained more phosphorus than corn tortillas. Tortillas with aluminum listed as a food additive contained higher aluminum concentrations than those without such listing, exceeding the tolerable weekly intake.

**Conclusions:**

Our study suggests that food additives contribute to the dietary burden of aluminum and phosphorus in ready-to-eat wheat flour tortillas. While additional investigations are warranted to confirm our findings, food reformulation strategies and clear food labeling will aid in reducing excess phosphorus and aluminum intake from processed foods.

## 1. Introduction

Chronic kidney disease (CKD) is associated with increased mortality due to an accumulation of metabolites and minerals that are normally excreted in the urine. These include urea, excess phosphorus and nonessential trace elements such as aluminum. Therefore, dietary restriction is a key component of renal disease management. Notably, bioaccumulation of minerals, including phosphorous and aluminum, is of particular concern due to inefficient clearance by dialysis (Martin & González, 2011; Pepper et al., 2011).

Hyperphosphatemia predicts adverse outcomes in renal patients (Chartsrisak et al., 2013). Phosphorus is well-known to be associated with cardiovascular disease and mortality in the general population as demonstrated in the NHANES III study (Chang et al., 2014). As the second most abundant mineral in the body, phosphorus plays a key role in essential biological processes including cell metabolism, acid-based balance, and bone calcification (Committee on Diet and Health; Food and Nutrition Board; Commission on Life Sciences; National Research Council, 1989). Hyperphosphatemia and secondary hyperparathyroidism are key contributors to bone disease, vascular calcification, and cardiovascular morbidity and mortality in CKD (Carrillo-López et al., 2019; Cozzolino et al., 2005). Therefore, dietary phosphorus restriction has been recommended for patients with cardiovascular disease or CKD (Gutiérrez, 2013; Takeda et al., 2007).

Elevated phosphate can be further complicated by aluminum toxicity. Aluminum intake is exacerbated by the administration of aluminum-based phosphate binders and supplementary agents such as citrate that enhance intestinal absorption of aluminum (Bohrer et al., 2008; Pepper et al., 2011). This can substantially contribute to aluminum bioavailability and accumulation in CKD patients (Bohrer et al., 2008; Pepper et al., 2011). The National Kidney Foundation recommends annual monitoring of serum aluminum levels in patients with CKD (Foundation, 2003). Efforts have been successfully implemented to address healthcare sources of aluminum such as the removal of aluminum from dialysis water. However, the dietary burden of aluminum remains unclear.

Grains are the top source of dietary phosphorus in the US diet followed by meat and milk products (McClure et al., 2017). Analysis of NHANES data from 2001 to 2014 indicated that while Mexican Americans consumed an amount of phosphorus comparable to non-Hispanic whites, a larger proportion of phosphorus in the Mexican American diet comes from grains containing a higher density of phosphorus including breads and Mexican mixed dishes (McClure et al., 2017). The high prevalence of diabetes and CKD among the Hispanic population have underlined the importance of dietary phosphate restriction in the Latin American diet (Aguayo-Mazzucato et al., 2019; Johnson et al., 2019; NIH, & NIDDK, 2023). Most notably, a staple food in the Mexican diet is the tortilla. Varying tortilla types and preparations can impact the phosphorus and aluminum levels. In 2001, the per capita intake of tortillas was 16.4 g per day in the US (Rooney et al., 2004), and this is likely higher in Mexican Americans as Hispanic adults are 2–3 times more likely to consume Mexican Food in the US compared to all other ethnic groups (Sebastian et al., 2010). Based on the data from the US Census Bureau, the demand for tortillas remains high in the US with a peak revenue remaining around $4 billion from 2012 to 2024 in tortillas manufacturing (US Census Bureau, 2022).

Tortillas are traditionally made fresh at home from flour derived from either corn or maize (Spanish *maíz*) or wheat (Spanish *harina;* Latin *farina*) (Saldivar & Chuck-Hernandez, 2016). The composition of tortillas vary depending on the ingredients used such as different types of corn varieties, thereby influencing their taste, texture, and nutritional content (Cabal-Prieto et al., 2024). However, like other bread products in the last century (Yokel, 2025), tortillas have become commercially available and are prepared with food additives that prolong shelf life and leavening agents to rise and soften the bread (Ficco et al., 2017; Vargas & Simsek, 2021). Notably, phosphorus-based additives are present in over 50 % of products from the top 25 US food and beverage manufacturers (Dunford & Calvo, 2025). This highlights the contribution of additives to the mineral content of commercially available tortillas, particularly the use of aluminum- and phosphorus- based agents (Ritz et al., 2012; Yokel & Florence, 2006). Under the Federal Food, Drug and Cosmetic Act, labelled food additives are Generally Recognized As Safe (GRAS) for consumption by the general public based on accepted scientific data and evaluation (U.S. Food and Drug Administration, 2023). However, this does not address the impact of food additives on renal patients who bioaccumulation minerals and are at a higher risk of toxicity. The true contribution of food additives to the dietary burden of phosphorus and aluminum in commercially-available corn and wheat flour tortillas has not been reported in the literature to our knowledge.

A single 6-inch corn and wheat tortillas lacking preservatives contains between 75 and 95 mg and 45–65 mg phosphorus, respectively, per 30 g serving (Davita, 2017). Dietary guidelines for CKD or dialysis patients have recommended limiting consumption of corn tortillas in favor of wheat flour tortillas (Davita, 2017), while remaining cautious of wheat flour tortillas containing phosphorus-based food additives (Gleason, 2022). We aim to elucidate the dietary aluminum and phosphorus burden in shelf-stable tortillas. We postulate that additives listed on food labels result in a significant amount of phosphorus and aluminum in ready-to-eat wheat flour or corn tortillas. This is of concern in CKD patients who bioaccumulate excess minerals including aluminum which can be fully excreted in the healthy population (Greger & Baier, 1983). Potential consequences of aluminum toxicity include neurological and neurodegenerative diseases such as dialysis dementia (Bondy, 2016; Igbokwe et al., 2019).

In accordance with the food label, we found that listed additives significantly contribute to the phosphorus and aluminum content of flour tortillas. The findings of the current study have implications for healthcare professionals managing CKD diets and for the food industry in reformulating additives to reduce nonessential trace elements such as aluminum.

## 2. Methods

Twenty-five different brands of tortillas, including 13 flour and 12 corn, available in local supermarkets in Southern California including Target, food4less, Walmart, and Ralph’s, as well as fast food chains including Del Taco and Taco Bell were purchased in October 2019 and March 2020. Each tortilla was assigned a code indicating whether it was corn- or wheat flour-based and hard or soft and was weighed. The weights (mean ± SD) of the soft corn, soft wheat, and hard corn tortillas were 27.7 ± 7.7, 42.0 ± 12.6, and 15.5 ± 1.4 g, respectively.

A sample weighing approximately 1 g was removed from each tortilla (1.14 ± 0.09 g, mean ± SD). A subsample weighing about 250 mg was removed, dried to a constant mass, accurately weighed, and digested using trace-metal grade HNO_3_ in sealed Teflon vessels in a CEM MARS Express microwave digestion system (U.S. EPA method 6020). Because homogenization occurs during the dough making process, we did not homogenize whole tortilla prior to digestion and the variance between replicates represents the composite variance within and between tortillas. The samples were analyzed by ICP-OES (Agilent 5110 SVDV; U.S. EPA Method 6010d) to quantify phosphorus with yttrium added as an internal standard. They were also analyzed by ICP-MS to quantify aluminum (Agilent 7900, Agilent Technologies, Inc., Santa Clarita, CA; U.S. EPA method 6020b) with germanium added as an internal standard. All analyzed samples were compared to NIST standard reference material 1515, Apple Leaves, to verify the accuracy of the methods. Instrument conditions are found in tables S1–S3.

Each tortilla was tested for 21 elements (Ag, Al, As, Ca, Cd, Co, Cr, Cu, Fe, K, Mg, Mn, Na, Ni, P, Pb, Se, Sr, U, V, and Zn) (Table S4, S5, and S7, Figure S1). Method detection limits (MDLs) are shown in Table S5. Food labels, either on the packaging or from online searches, were compiled to identify the ingredients, focusing on food additives comprising phosphorus and aluminum. Multiple group comparisons were analyzed by one-way ANOVA with Tukey’s post hoc test. Outliers were identified using the ROUT test on GraphPad/PRISM and statistical significance was determined using nonparametric Mann-Whitney and Kruskal-Wallis tests. All conditions (soft corn, hard corn, and wheat) were compared using Dunn’s multiple comparison test (Table S6 and S8).

### Justification for Sample Size:

There are limited commercial suppliers of ready-to-eat tortillas, thereby limiting the sample size of this study. An a priori power analysis was therefore conducted to determine whether the achievable sample size was sufficient to detect meaningful differences between product types. Assuming a two-sided comparison between corn (n = 12) and flour (n = 13) tortillas, a significance level of α = 0.05 (95 % confidence), and a large effect size (Cohen’s d ≥ 0.8), the resulting total sample size of 25 provided approximately 80 % statistical power to detect differences in elemental concentrations. The assumption of a large effect size was considered appropriate given the expected formulation and ingredient differences among commercial tortilla brands (Cohen, 1988). Statistical power calculations were performed using WebPower (WebPower, 2018). Most samples were analyzed in duplicate from the same tortilla package to assess analytical consistency. To account for variation in tortilla size across brands, all measurements were standardized and expressed per 30-g serving. Given the exploratory and comparative nature of this study and the limited number of commercially available products, the study was powered to detect large, practically meaningful differences rather than small effects.

This study was deemed exempt by the University of Kentucky Institutional Review Board. This study was deemed exempt under federal regulation 45 46.101 (b) CFR.(US Department of Health and Human Services, 2025)

## 3. Results

**Fig. 1.**
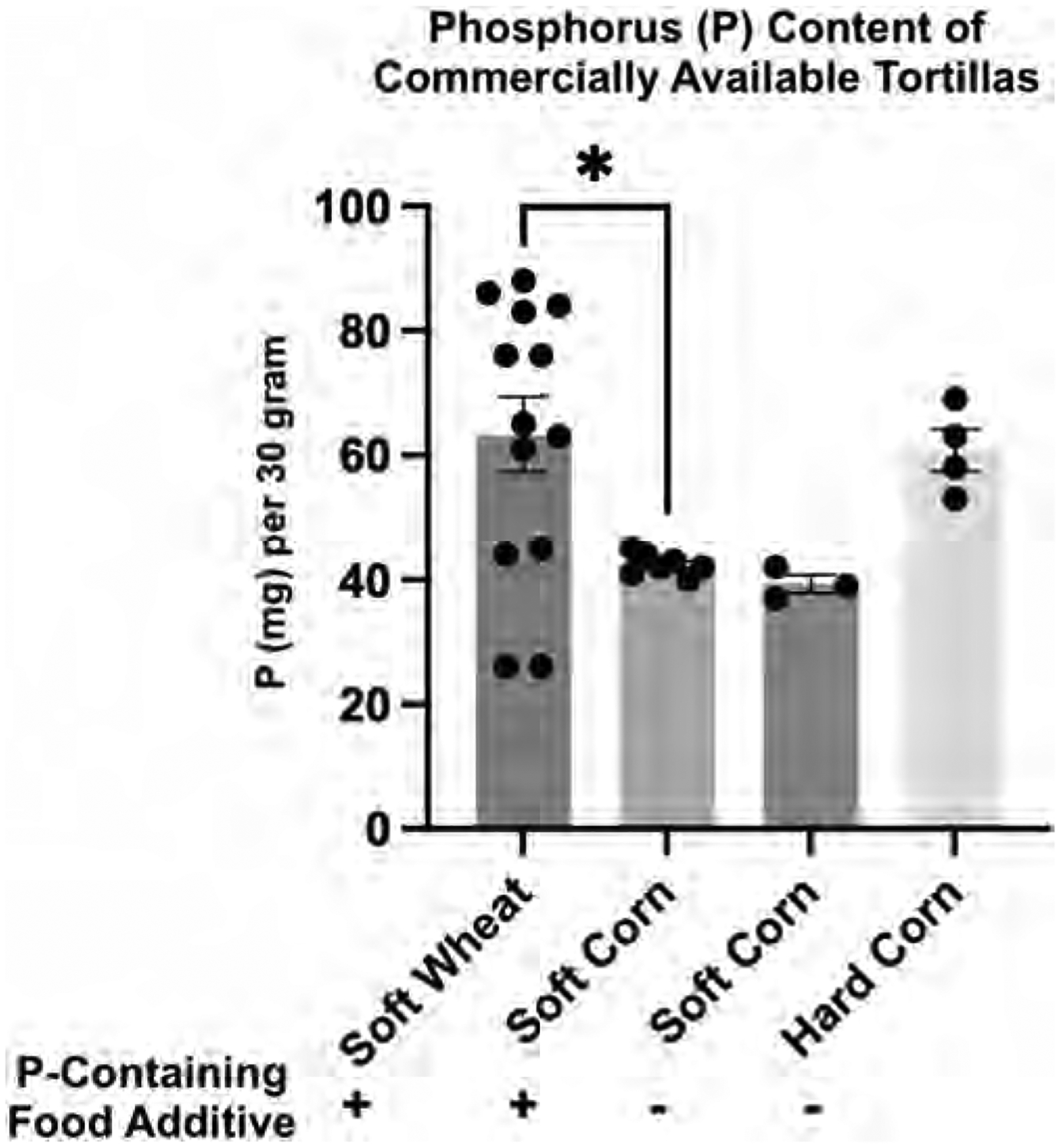
Total elemental phosphorus (P) content (mg P/30 g serving) of commercially available, ready-to-eat tortillas, with or without P-containing food additives as per the label, including soft wheat, soft corn, and hard corn tortillas. *P < 0.05; ANOVA p-value = 0.026; d = 0.89. Values are mean ± SEM.

The elemental content of store-bought, ready-to-eat, wheat and corn tortillas was quantified. The elemental concentrations measured in this study were comparable to the sodium, calcium, iron, and potassium concentrations on the package label (Table S9). Further analysis of phosphorus concentrations reveals that wheat flour tortillas generally contained approximately 50 % more phosphorus (per 30 grams serving) than corn tortillas when a source of phosphorus was listed on the ingredients label (p = 0.049). This resulted in an effect size of 0.74, indicating practical significance between the two (Fig. 1). Notably, wheat flour tortillas from the brands Calidad, Great Value, La Banderita, and Old El Paso contained approximately double the average phosphorus content of corn tortillas, even when the latter listed phosphorus containing additives on the label (Table 1).

**Fig. 2.**
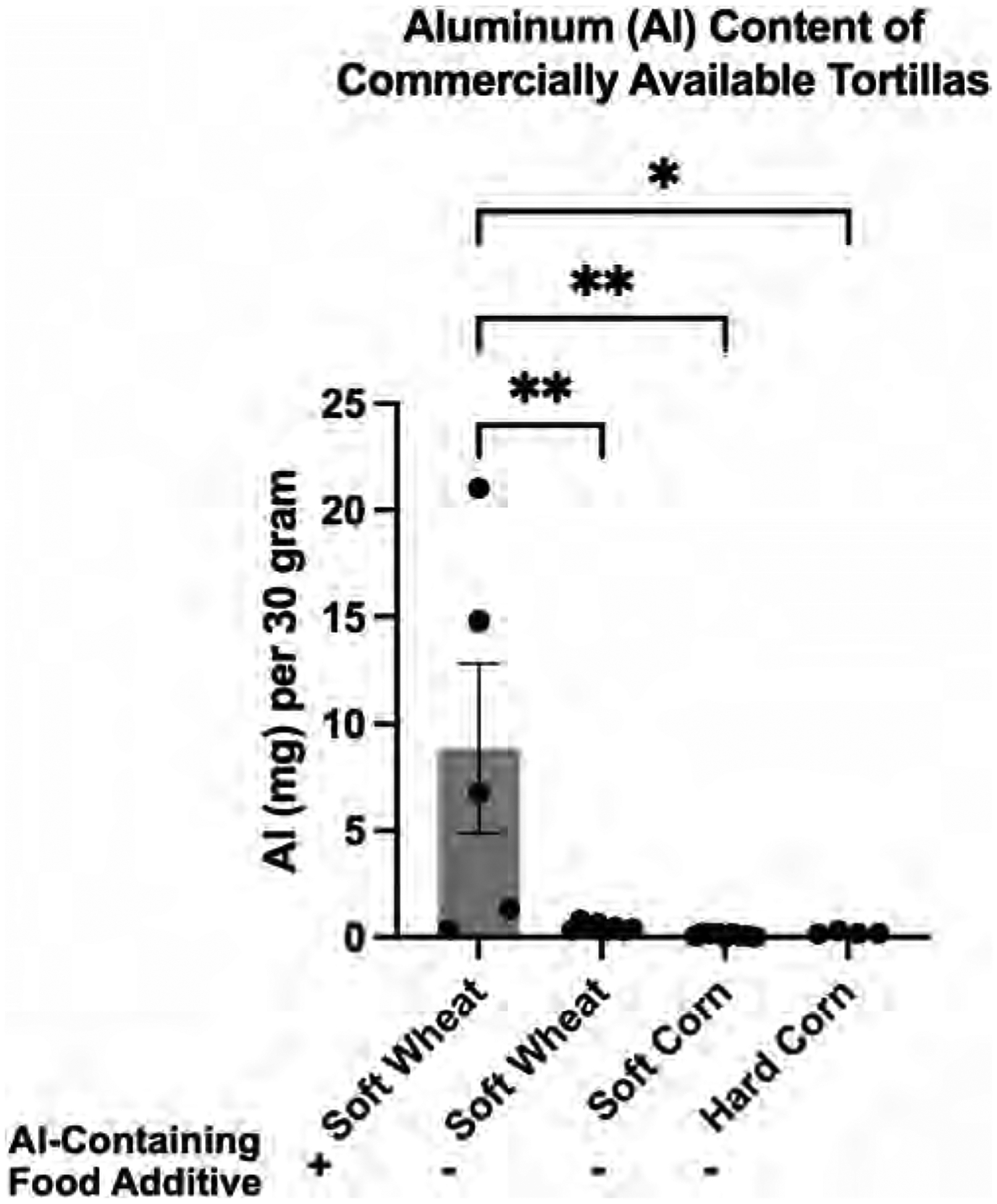
Aluminum (Al) content (mg Al/30 g serving) of commercially available, ready-to-eat tortillas, with or without Al-containing food additives as listed on the label, including soft wheat, soft corn, and hard corn tortillas. *P < 0.05 **P < 0.01; ANOVA p-value = 0.0013; d = 0.75. Wheat flour values are mean ± SEM.

The tested brands of corn tortillas had low aluminum content consistent with the lack of aluminum-containing food additives reported on the label (Fig. 2). On the other hand, wheat flour tortillas with reported aluminum additives had markedly elevated amounts of aluminum per 30 gram serving (p = 0.0032 and p = 0.0015 compared to wheat flour and corn tortillas with no added aluminum) (Fig. 2). The effect sizes were 0.64, 0.69, and 0.56 when compared to wheat without aluminum additives, soft corn, and hard corn tortillas, respectively. This suggests that in wheat tortillas containing added aluminum, the aluminum content is significantly higher compared to the other tortilla products. While the levels of aluminum may partially overlap between brands with and without listed aluminum (Table 2), wheat flour tortillas generally contained more aluminum than corn tortillas even in the absence of listed aluminum additives (Table S6).

Four brands of hard corn tortillas were studied: Del Taco, La Pericos, Old El Paso, and Taco Bell (Table S5). The label on all four did not list any phosphorus- or aluminum-containing food additives. The phosphorus content ranged from 53 to 69 mg per 30 gram serving. This was comparable to wheat flour tortillas with phosphorus additives (63 ± 6.0 mg per 30 g serving; mean ± SEM) (Table 1). The aluminum content ranged from 0.15 to 0.31 mg per 30 g serving, less than wheat flour tortillas with aluminum additives (8.86 ± 3.98 mg per 30 g serving; mean ± SEM) (Table 2) and comparable to soft corn tortillas (0.02–0.45 mg per 30 g serving, Table S4).

Further analysis of additional elements revealed that wheat flour tortillas generally contained higher sodium concentrations compared to corn tortillas regardless of sodium-containing food additives (Tables S4, S5, and S7 in Supplemental File 1).

## 4. Discussion

This study describes the phosphorus and aluminum content of ready-to-eat tortillas obtained from supermarkets and food franchises in Southern California. Our findings demonstrate that wheat flour tortillas with approved food additives can have a phosphorus and aluminum content of up to about 90 mg and 20 mg per 30 g serving, respectively. When elemental content is analyzed per tortilla, wheat flour consistently has significantly higher levels of phosphorus and aluminum compared to corn tortillas (p = 0.005 and <0.0001, respectively) (Table S6). These findings challenge the previous assumption that consumption of corn tortillas should be avoided or restricted in patients with CKD due to the intrinsically high phosphorus content of corn (Davita Kidney Care, 2022). According to data from Davita Kidney Care, the phosphorus content for a 30 g portion of a six-inch corn tortilla is 75 mg while a wheat flour tortilla made without baking powder is 20–37 mg (Davita Kidney Care, 2022). However, most shelf-stable flour tortillas contain phosphorus-based food additives that can increase phosphorus concentrations above levels found in corn tortillas. These findings are of great public health importance, especially for countries with a large Hispanic population.

Elevated phosphorus and aluminum levels are consistent with the preservatives listed on the packaging. The following preservatives were commonly used: calcium propionate, propionic acid, sorbic acid, potassium sorbate, fumaric acid, citric acid, benzoic acid, phosphoric acid, lime, and sodium hydroxide. Additives such as phosphoric acid, sodium aluminum phosphate, and sodium acid pyrophosphate may have contributed to higher levels of phosphorus in wheat flour compared to corn tortillas.

The daily dietary intake of an adult in the US comprises about 5 mg aluminum (Greger, 1992; Yokel, 2023, 2025). The per capita tortilla consumption is about 230–330 g per day as reported from Mexico (Rooney et al., 2004). Based on an average intake of 8.9 mg of aluminum per 30 g serving of a wheat flour tortilla found in the current study, the daily aluminum exposure would be about 28–40 mg of aluminum. The Food and Agriculture Organization of the United Nations and the WHO established a provisional tolerable weekly intake (PTWI) of 2 mg/kg body weight that applies to all aluminum compounds in food (World Health Organization, 2011). The European Food Safety Authority established a tolerable weekly intake of 1 mg/kg body weight (European Food Safety Authority (EFSA), 2008). The U.S. Agency for Toxic Substances and Disease Registry minimal risk level for oral aluminum for 15 days or longer exposure is 1 mg/kg/day (European Food Safety Authority (European Food Safety Authority EFSA, 2008). With a projected annual growth rate of 3.4 %, the global commercial tortilla market may grow from 26 billion in 2022 to approximately 37 billion in 2032 (Future Market Insights, 2022). Therefore, the rising popularity of tortillas may potentially increase aluminum exposure above the PTWI and may pose a serious health risk to patients with CKD.

Even though not all tested brands listed aluminum on the label, we found that the range of aluminum content of wheat flour tortillas without added aluminum (0.31–14.83) overlaps with those that have added aluminum (0.31–21.04). Furthermore, wheat flour tortillas contained markedly higher levels of sodium compared to corn tortillas (Table S4 and S7). Excess aluminum and sodium in wheat flour tortillas may be incorporated during the preparation process or present in the baking ingredients. Sodium bicarbonate (baking soda), monocalcium phosphate, and either sodium acid pyrophosphate or sodium aluminum sulfate are often used as additives or preservatives in commercially-available tortillas (NC State University, 2014). Baking powder is commonly used as a leavening agent in making bread and can be a source of aluminum (Saiyed & Yokel, 2005). Wheat flour tortillas contain about 0.02–3.75 mg aluminum per 30 g serving depending on its source (University of Kentucky, 2025; Yokel, 2025). While wheat flour has been reported to contain a higher aluminum concentration compared to corn flour (Brizio et al., 2016; Squadrone et al., 2021), lower levels in wheat flour have also been observed (Rubio-Armendáriz et al., 2021). Manufacturers should consider reformulation strategies, modifying the preparation process and actively monitoring product composition to account for excess minerals beyond the listed ingredients. For example, alternatives to phosphorus- and aluminum-containing commercial baking powders include biological leavening agents such as yeast, leavening acids, and mechanical leavening with steam (Gélinas, 2021, 2022).

The cereal and grain food groups mainly comprising corn and wheat flour tortillas, respectively, make up approximately 40 % of the total energy intake of low-income Mexican agricultural workers (Castañeda et al., 2019). The annual per capita consumption of tortillas in the US and Mexico in 2001 was 6 kg and 85 kg, respectively (Rooney & Serna-Saldivar, 2016). Hispanics in the US are 2–3 times more likely to consume Mexican food than other ethnic groups (Sebastian et al., 2010), and are therefore likely to consume more tortillas than the average per capita intake in the US. Given its popularity in making up a significant proportion of the Mexican diet, some soft flour tortillas, e.g., Del Taco, Old El Paso, Romero’s, may lead to aluminum consumption that exceeds TWI of 1.0 mg Al/kg body weight (Yokel, 2023). High rates of CKD of unknown origin have been frequently reported in agricultural workers known as mesoamerican nephropathy (Johnson et al., 2019). Interestingly, toenail aluminum concentration was almost twice as high in those with acute Mesoamerican nephropathy than controls (Fischer et al., 2020). While further research is needed on the consumption of store-bought tortillas by Central American workers with Mesoamerican nephropathy, this staple food may contribute to aluminum toxicity and phosphorus associated cardiovascular disease.

Limitations of the current study include a small sample size that does not account for the diversity of tortilla brands globally and the different local preparation techniques for homemade tortillas. Further studies are needed to assess the aluminum and phosphorus content of homemade tortillas. While this study focuses on the most traditionally consumed tortillas, testing other preparations such as gluten-free, low-carb and high-protein tortillas may also reveal discrepancies in the burden of aluminum and phosphorus. Moreover, directly measuring the impact of tortilla intake on the aluminum and phosphorus levels in patients with kidney and cardiovascular disease would further guide dietary recommendations.

The elemental concentrations measured in the current study were overall consistent with the available calcium, iron, potassium, and sodium levels reported on the packaging and ingredient labeling (Table S9). Our results indicate that patients and healthcare providers should be cautious of food labeling and limit intake of products with listed phosphorus and aluminum-based additives, particularly wheat flour tortillas. In addition to reformulation strategies, more stringent package labeling that highlights the nutritional and complete mineral content of packaged foods will encourage renal-friendly manufacturing practices and aid in the management of the CKD diet.

## 5. Conclusion

Ready-to-eat wheat flour tortillas have a higher elemental phosphorus concentration than corn (maize) tortillas due to added preservatives and leavening agents. Use of aluminum-based leavening agents to make ready-to-eat wheat flour tortillas confers a higher aluminum concentration compared to corn tortillas. The aluminum and phosphorus concentrations of ready-to-eat tortilla brands can present a daily dietary load exceeding 100 mg of aluminum and up to 700 mg of phosphorus, based on an average daily tortilla intake of 330 grams. Therefore, these findings confirm that food additives contribute to a higher aluminum and phosphorus content in ready-to-eat wheat flour tortillas. Patients with CKD should favor homemade rather than ready-to-eat tortillas and pay close attention to additives listed on the label. This would be facilitated by more stringent food labeling highlighting potential sources of aluminum on food packaging. Tortilla manufacturers should also consider reformulation strategies to reduce the dietary burden of phosphorus and aluminum from food additives and from incidental incorporation during the preparation process.

## Supplementary Material

Supplementary file 2

Supplementary file 3

Supplementary file 1

## Figures and Tables

**Table 1:** Phosphorus (P) content of wheat flour and corn tortillas with phosphorus-containing food additives as per the label.

	Brand	P-containing food additives	P (mg) per 30 g serving^
**Wheat Flour Tortillas**	Calidad (n = 2)	Na_2_H_2_P_2_O_7_, Ca (H_2_PO_4_)_2_	83
	Diana’s (n = 2)	Al_3_H_22_NaO_36_P_8_	26
	Del Taco (n = 2)	Na_2_H_2_P_2_O_7_, Ca (H_2_PO_4_)_2_	26
	El Comal (n = 2)	Na_2_H_2_P_2_O_7_, Ca (H_2_PO_4_)_2_	63
	Guerrero (n = 2)	Na_2_H_2_P_2_O_7_	44
	Great Value (n = 2)	Na_2_H_2_P_2_O_7_	86
	Kroger (n = 2)	Na_2_H_2_P_2_O_7_	76
	La Banderita (n = 2)	Na_2_H_2_P_2_O_7_, Ca (H_2_PO_4_)_2_	84
	Mission (n = 2)	Na_2_H_2_P_2_O_7_	65
	Más y Más (n = 2)	Na_2_H_2_P_2_O_7_	76
	Old El Paso (n = 2)	Al_3_H_22_NaO_36_P_8_	88
	Romero’s (n = 2)	Na_2_H_2_P_2_O_7_	45
	Taco Bell (n = 1)	Na_2_H_2_P_2_O_7_, CaHPO_4_	61
	**Mean (SEM)**	63 (6)	
**Corn Tortillas**	Calidad (n = 2)	H_3_PO_4_	42
	Del Taco (n = 2)	H_3_PO_4_	45
	El Comal (n = 2)	H_3_PO_4_	40
	Guerrero (n = 1)	H_3_PO_4_	43
	Great Value (n = 1)	H_3_PO_4_	44
	Mission (n = 2)	H_3_PO_4_	41
	Romero’s (n = 1)	H_3_PO_4_	42
	**Mean (SEM)**	42 (0.6)	
**Flour vs Corn Tortillas**	**Ratio (flour/corn)**	1.49	
	**% increase** *	50 %	

^ This is the average value of duplicate measurements on each sample P refers to inorganic phosphorus, including P in the grain.

* [(P in flour-P in corn)/P in corn] × 100

**Table 2:** Aluminum content of wheat flour tortillas with or without aluminum-containing food additives as per the label.

	Brand	Al-containing food additives	Al (mg) per 30 g serving^
**Al-containing food additives added as per label**	Calidad (n = 2)	NaAl(SO_4_)_2_	0.35
	Diana’s (n = 2)	NaAl(SO_4_)_2_ Al_3_H_22_NaO_36_P_8_	1.33
	Del Taco (n = 2)	NaAl(SO_4_)_2_	14.83
	Old El Paso (n = 2)	Al_3_H_22_NaO_36_P_8_	21.04
	Romero’ s (n = 2)	NaAl(SO_4_)_2_	6.77
	**Mean (SEM)**	8.86 (3.98)	
**Aluminum Ingredient-free as per label**	El Comal (n = 2)	-	0.65
	Guerrero (n = 2)	-	0.31
	Great Value (n = 2)	-	0.47
	Kroger (n = 2)	-	0.41
	La Banderita (n = 2)	-	0.31
	Mission (n = 2)	-	0.33
	Más y Más (n = 2)	-	0.43
	Taco Bell (n = 1)	-	0.80
	**Mean (SEM)**	0.46 (0.06)	
**Al added vs Al not added** **	**Ratio (Al added/Al not added)**	~19	
	**% increase** ^+^	1800 %	

^ This is the average value of duplicate measurements on each sample

+ [(Al added-Al not added)/Al not added] × 100

** As per the ingredients listed by the manufacturer in the label

## Research question

Dietary restrictions, including on tortilla consumption, aim to limit phosphorus intake in patients with renal disease. Aluminum clearance is reduced in renal disease. Given the dietary burden of food additives, do food additives substantially contribute to the phosphorous and aluminum content of commercially available wheat flour and corn tortillas?

## Key findings

In this cross-sectional analysis of 14 corn and 13 wheat flour tortilla brands purchased from local supermarkets in Southern California from 2019 to 2020, ready-to-eat wheat flour tortillas consistently had a higher phosphorus content compared to corn tortillas due to food additives. Wheat flour tortillas also had a higher aluminum content, consistent with listed aluminum-based additives.

## Appendix A. Supporting information

Supplementary data associated with this article can be found in the online version at doi:10.1016/j.foohum.2026.101060.

## Abbreviations:

P: Phosphorus
Al: aluminum
CKD: chronic kidney disease
TWI: tolerable weekly intake
ICP-MS: inductively coupled plasma mass spectrometry
ICP-OES: inductively coupled plasma optical emission spectroscopy
