# Supplementary material for: The dietary burden of phosphorus and aluminum in ready-to-eat wheat flour tortillas exceeds that of corn tortillas: Implications for patients with chronic kidney disease
Source: Food Humanit. Author manuscript; Available in PMC 2026 Jul 23. (PMC13390896; doi:10.1016/j.foohum.2026.101060)
Supplement: Supplementary file 2 [file NIHMS2196054-supplement-Supplementary_file_2.docx]

Table S1 – Wavelengths used for quantification in inductively coupled plasma optical emission spectrometry (ICP-OES).

| **Element Symbol** | **Wavelength (nm)** |
| --- | --- |
| Ca | 422.673 |
| Fe | 238.204 |
| K | 766.491 |
| Mg | 279.553 |
| Na | 589.592 |
| P | 213.618 |

Table S2 - Mass to charge ratios (m/z) used for quantification in inductively coupled plasma mass spectrometry (ICP-MS).

| **Element** | **m/z** |
| --- | --- |
| Ag | 107 |
| Al | 27 |
| As | 75 |
| Cd | 111 |
| Co | 59 |
| Cr | 52 |
| Cu | 65 |
| Mn | 55 |
| Ni | 60 |
| Pb | 208 |
| Se | 78 |
| Sr | 88 |
| U | 238 |
| V | 51 |
| Zn | 66 |

Table S3. Operating conditions used for inductively coupled plasma mass spectrometry (ICP-MS) and inductively coupled plasma optical emission spectrometry (ICP-OES).

| **Parameter** | **ICP-MS** | **ICP-OES** |
| --- | --- | --- |
| Nebulizer | Micromist, 0.2 ml/min | Seaspray, 1 mL/min |
| Spray chamber | Scott, cooled to 4°C | Cyclonic, room temp |
| RF power (W) | 1550 | 1200 |
| Operating mode | no gas, He (As, Cr, Cu, Zn), H_2_ (Se) | simultaneous dual view |
| Nebulizer gas flow (L/min) | 1.05 | 0.7 |
| Plasma gas flow (L/min) | 15 | 12 |
| Auxilliary gas flow (L/min) | 0.9 | 1 |

**Table S4. Elemental concentrations in soft corn tortillas**

| **Source** | Calidad (n=2) | Del Taco (n=2) | El Comal (n=2) | El Milagro (n=1) | Guerrero (n=1) | Great Value (n=1) | La Banderita (n=1) | Mission (n=2) | Romero's (n=1) | Taco Bell (n=1) |
| --- | --- | --- | --- | --- | --- | --- | --- | --- | --- | --- |
| **Hard or Soft** | S | S | S | S | S | S | S | S | S | S |
| **Total Tortilla Weight (g) ^1^** | 22.64 | 26.73 | 36.91 | 24.57 | 24.00 | 22.44 | 24.64 | 23.52 | 25.51 | 45.98 |
| P (mg/30g) | 42.46 | 44.70 | 39.67 | 37.05 | 43.02 | 43.55 | 39.30 | 40.71 | 41.56 | 41.60 |
| Al (mg/30g) | 0.21 | 0.15 | 0.17 | 0.08 | 0.02 | 0.06 | 0.02 | 0.19 | 0.06 | 0.45 |
| Ca (mg/30g) | 11.57 | 11.37 | 35.27 | 60.08 | 9.84 | 10.52 | 8.85 | 9.72 | 11.35 | 36.37 |
| Co (mg/30g) | 0.00 | 0.00 | 0.00 | 0.00 | 0.00 | 0.00 | 0.00 | 0.00 | 0.00 | BDL |
| Cr (mg/30g) | 0.00 | 0.00 | 0.00 | 0.00 | 0.00 | 0.00 | 0.00 | 0.00 | 0.00 | 0.01 |
| Cu (mg/30g) | 0.03 | 0.03 | 0.03 | 0.03 | 0.03 | 0.03 | 0.03 | 0.03 | 0.03 | 0.07 |
| Fe (mg/30g) | 0.44 | 0.21 | 0.23 | 0.24 | 0.18 | 0.24 | 0.17 | 0.20 | 0.30 | 0.90 |
| K (mg/30g) | 46.37 | 48.84 | 41.62 | 34.97 | 48.48 | 48.81 | 47.42 | 44.98 | 46.59 | 36.55 |
| Mg (mg/30g) | 16.33 | 17.21 | 13.23 | 14.23 | 15.51 | 15.86 | 15.42 | 15.27 | 16.38 | 9.72 |
| Mn (mg/30g) | 0.09 | 0.09 | 0.08 | 0.08 | 0.11 | 0.10 | 0.10 | 0.09 | 0.10 | 0.25 |
| Na (mg/30g) | 10.56 | 10.83 | 3.75 | 0.31 | 13.02 | 11.92 | 11.53 | 11.21 | 8.42 | 150.18 |
| Ni (mg/30g) | 0.01 | 0.01 | 0.01 | 0.01 | 0.01 | 0.01 | 0.01 | 0.01 | 0.01 | 0.01 |
| Se (mg/30g) | 0.00 | 0.00 | 0.00 | BDL | 0.00 | BDL | 0.00 | 0.00 | BDL | 0.01 |
| Sr (mg/30g) | 0.03 | 0.03 | 0.04 | 0.04 | 0.03 | 0.03 | 0.03 | 0.03 | 0.03 | 0.06 |
| Zn (mg/30g) | 0.43 | 0.49 | 0.36 | 0.39 | 0.44 | 0.46 | 0.43 | 0.40 | 0.45 | 0.89 |
|  |  |  |  |  |  |  |  |  |  |  |
| **Pi additive** | Pi additive |  | Pi additive |  | Pi additive | Pi additive |  | Pi additive | Pi additive |  |
| **P (mg/tortilla)** | 32 | 40 | 49 | 30 | 34 | 33 | 32 | 32 | 35 | 64 |
| **P (mg/30 g serving)** | 42 | 45 | 40 | 37 | 43 | 44 | 39 | 41 | 42 | 42 |
| **Al additive** |  |  |  |  |  |  |  |  |  |  |
| **Al (mg/tortilla)** | 0.16 | 0.14 | 0.20 | 0.07 | 0.02 | 0.04 | 0.02 | 0.15 | 0.05 | 0.69 |
| **Al (mg/30 g serving)** | 0.21 | 0.15 | 0.17 | 0.08 | 0.02 | 0.06 | 0.02 | 0.19 | 0.06 | 0.45 |

^1^ Tortilla weight and element concentrations are averages of one or two tortillas (noted in the source).

Ag was below the MDL (0.0010) in all samples. As was ≤ 0.02 in all samples. Cd was ≤ 0.01 in corn and ≤ 0.03 in flour samples. Pb was ≤ 0.05 in all samples. U was ≤ 0.02 in all samples. V was ≤ 0.05 in all samples.

Outliers highlighted in red

**Table S5. Elemental concentrations in hard corn tortillas**

| **Source** | Del Taco (n=2) | La Pericos (n=2) | Old El Paso (n=2) | Taco Bell (n=1) | MDL (mg/30g |
| --- | --- | --- | --- | --- | --- |
| **Hard or Soft** | H | H | H | H |  |
| **Total Tortilla Weight (g) ^1^** | 15.65 | 14.08 | 14.90 | 17.35 |  |
| P (mg/30g) | 68.89 | 62.86 | 53.08 | 57.74 | 0.06 |
| Al (mg/30g) | 0.15 | 0.17 | 0.17 | 0.31 | 0.00 |
| Ca (mg/30g) | 51.49 | 18.96 | 22.86 | 13.72 | 0.12 |
| Co (mg/30g) | 0.00 | 0.00 | 0.00 | BDL | 0.00 |
| Cr (mg/30g) | 0.01 | 0.00 | 0.00 | 0.00 | 0.00 |
| Cu (mg/30g) | 0.05 | 0.05 | 0.05 | 0.05 | 0.00 |
| Fe (mg/30g) | 0.55 | 0.39 | 0.28 | 0.18 | 0.12 |
| K (mg/30g) | 69.05 | 58.71 | 55.77 | 63.80 | 0.12 |
| Mg (mg/30g) | 23.95 | 22.20 | 17.57 | 23.88 | 0.06 |
| Mn (mg/30g) | 0.16 | 0.14 | 0.13 | 0.16 | 0.00 |
| Na (mg/30g) | 143.38 | 140.30 | 105.72 | 1.25 | 0.12 |
| Ni (mg/30g) | 0.01 | 0.01 | 0.01 | 0.02 | 0.00 |
| Se (mg/30g) | 0.00 | 0.00 | 0.00 | 0.01 | 0.00 |
| Sr (mg/30g) | 0.05 | 0.03 | 0.05 | 0.05 | 0.00 |
| Zn (mg/30g) | 0.69 | 0.70 | 0.62 | 0.63 | 0.04 |
|  |  |  |  |  |  |
| **Pi additive** | Pi additive |  |  |  |  |
| **P (mg/tortilla)** | 36 | 30 | 26 | 33 |  |
| **P (mg/30 g serving)** | 69 | 63 | 53 | 58 |  |
| **Al additive** |  |  |  |  |  |
| **Al (mg/tortilla)** | 0.08 | 0.08 | 0.08 | 0.18 |  |
| **Al (mg/30 g serving)** | 0.15 | 0.17 | 0.17 | 0.31 |  |

^1^ Tortilla weight and element concentrations are averages of one or two tortillas (noted in the source).

Ag was below the MDL (0.0010) in all samples. As was ≤ 0.02 in all samples. Cd was ≤ 0.01 in corn and ≤ 0.03 in flour samples. Pb was ≤ 0.05 in all samples. U was ≤ 0.02 in all samples. V was ≤ 0.05 in all samples.

Outliers highlighted in red

**Table S6. Average P and Al concentration in soft and hard corn tortillas**

|  | **Total Tortilla Weight (g) ^1^** | **P (mg/kg)** | **Al (mg/kg)** | **P (mg/tortilla)** | **P (mg/30 g serving)** | **Al (mg/tortilla)** | **Al (mg/30 g serving)** |
| --- | --- | --- | --- | --- | --- | --- | --- |
| average (mean) all results | 24.2 | 1562 | 5.23 | 36.2 | 46.9 | 0.14 | 0.16 |
| median all results | 23.8 | 1425 | 5.28 | 33 | 42.7 | 0.08 | 0.16 |
| average (mean) non-outliers | 24.2 | 1506 | 5.23 | 32.8 | 45.2 | 0.1 | 0.16 |
| median non-outliers | 23.8 | 1415 | 5.28 | 32.4 | 42.5 | 0.08 | 0.16 |
| are all data normally distributed? | no | no | yes | no | no | no | yes |
| all data unpaired two-tailed Mann Whitney test P value (corn vs wheat) | 0.0002 | 0.0174 | <0.0001 | 0.0005 | 0.017 | < 0.0001 | <0.0001 |
| are non-outliers normally distributed? | no | no | yes | yes | no | yes | yes |
| non-outliers unpaired two-tailed Mann Whitney test P value (corn vs wheat) | 0.0002 | 0.0106 | <0.0001 | 0 | 0 | <0.0001 | <0.0001 |
|  |  |  |  |  |  |  |  |
| average (mean) all hard tortilla results | 15.5 | 2021 | 6.61 | 31.3 | 60.6 | 0.1 | 0.2 |
| median all hard tortilla results | 15.3 | 2010 | 5.52 | 31.5 | 60.3 | 0.08 | 0.17 |
| average (mean) non-outliers hard tortilla results | 15.5 | 1930 | 6.61 | 31.3 | 57.9 | 0.1 | 0.2 |
| average (mean) all soft tortilla results | 27.7 | 1379 | 4.25 | 38.1 | 41.4 | 0.15 | 0.14 |
| median all soft tortilla results | 24.6 | 1386 | 3.88 | 33.5 | 41.6 | 0.1 | 0.12 |
| average (mean) non-outlier soft tortilla results | 27.7 | 1379 | 4.67 | 38.1335051 | 41.3635868 | 0.15268282 | 0.14023735 |
| Kruskall-Wallis test of non-outlier soft corn, hard corn, and wheat, P value | 0.0003 | 0.0085 | 0.0002 | 0.0033 | 0.0091 | 0.0009 | 0.0009 |

| Stat tests results below of Dunn's multiple comparison's test, after Kruskal-Wallis test |
| --- |
| Total weight: soft * and hard *** different from wheat |
| P (mg/kg) soft corn different from wheat ** |
| Al (mg/kg) soft *** and hard *** corn different from wheat |
| P mg/tortilla soft* and hard* corn different from wheat |
| P mg/30 g soft ** different from wheat |
| Al mg/tortilla soft ** and hard * different from wheat |
| Al mg/serving soft *** different from wheat |
|  |
| ^1^ Tortilla weight and element concentrations are averages of one or two tortillas (noted in the source). |

**Table S7. Elemental concentrations in flour tortillas**

| **Source** | Calidad (n=2) | Diana’s (n=2) | Del Taco (n=2) | El Comal (n=2) | Guerrero (n=2) | Great Value (n=2) | Kroger (n=2) | La Banderita (n=2) | Mission (n=2) | Mas y Mas (n=2) | Old El Paso (n=2) | Romero (n=2) | Taco Bell (n=1) |
| --- | --- | --- | --- | --- | --- | --- | --- | --- | --- | --- | --- | --- | --- |
| **Hard or Soft** | S | S | S | S | S | S | S | S | S | S | S | S | S |
| **Total Tortilla Weight (g) ^1^** | 41.63 | 39.80 | 26.04 | 37.99 | 39.31 | 49.57 | 50.19 | 56.41 | 70.18 | 45.15 | 31.94 | 33.52 | 24.07 |
| P (mg/30g) | 82.92 | 25.85 | 26.38 | 62.82 | 44.03 | 86.32 | 76.39 | 83.70 | 65.10 | 76.22 | 87.67 | 44.71 | 61.19 |
| Al (mg/30g) | 0.35 | 1.33 | 14.83 | 0.65 | 0.31 | 0.47 | 0.41 | 0.31 | 0.33 | 0.43 | 21.04 | 6.77 | 0.80 |
| Ca (mg/30g) | 74.56 | 54.69 | 69.98 | 41.79 | 26.11 | 67.37 | 67.44 | 44.65 | 55.33 | 68.51 | 31.08 | 53.47 | 62.05 |
| Co (mg/30g) | 0.00 | 0.00 | 0.00 | 0.00 | 0.00 | 0.00 | 0.00 | 0.00 | 0.01 | 0.00 | 0.00 | 0.00 | BDL |
| Cr (mg/30g) | 0.00 | 0.00 | 0.01 | 0.00 | 0.00 | 0.00 | 0.00 | 0.00 | 0.01 | 0.00 | 0.00 | 0.01 | 0.00 |
| Cu (mg/30g) | 0.04 | 0.04 | 0.05 | 0.04 | 0.04 | 0.04 | 0.04 | 0.06 | 0.04 | 0.06 | 0.04 | 0.04 | 0.06 |
| Fe (mg/30g) | 1.15 | 0.89 | 1.00 | 0.24 | 0.81 | 0.22 | 1.06 | 1.50 | 1.20 | 0.99 | 0.66 | 0.82 | 1.19 |
| K (mg/30g) | 29.45 | 25.84 | 27.27 | 40.09 | 26.39 | 26.45 | 26.89 | 26.23 | 26.55 | 27.00 | 59.26 | 38.90 | 34.09 |
| Mg (mg/30g) | 6.19 | 5.97 | 6.13 | 6.45 | 5.88 | 6.19 | 6.16 | 7.38 | 6.22 | 5.97 | 6.35 | 7.06 | 8.18 |
| Mn (mg/30g) | 0.24 | 0.20 | 0.21 | 0.22 | 0.21 | 0.22 | 0.22 | 0.25 | 0.22 | 0.23 | 0.24 | 0.21 | 0.25 |
| Na (mg/30g) | 269.65 | 133.54 | 219.88 | 189.50 | 158.67 | 237.33 | 243.51 | 216.57 | 251.61 | 241.66 | 172.12 | 176.83 | 260.63 |
| Ni (mg/30g) | 0.01 | 0.01 | 0.01 | 0.00 | 0.00 | 0.01 | 0.00 | 0.01 | 0.00 | 0.00 | 0.00 | 0.01 | 0.01 |
| Se (mg/30g) | 0.01 | 0.01 | 0.01 | 0.01 | 0.01 | 0.01 | 0.00 | 0.01 | 0.00 | 0.01 | 0.00 | 0.01 | 0.01 |
| Sr (mg/30g) | 0.07 | 0.07 | 0.12 | 0.05 | 0.04 | 0.07 | 0.06 | 0.06 | 0.05 | 0.07 | 0.04 | 0.06 | 0.07 |
| Zn (mg/30g) | 0.25 | 0.31 | 0.26 | 0.30 | 0.26 | 0.28 | 0.22 | 0.32 | 0.28 | 0.32 | 0.37 | 0.32 | 0.37 |
|  |  |  |  |  |  |  |  |  |  |  |  |  |  |
| **Pi additive** | Pi additive | Pi additive | Pi additive | Pi additive | Pi additive | Pi additive | Pi additive | Pi additive | Pi additive | Pi additive | Pi additive | Pi additive | Pi additive |
| **P (mg/tortilla)** | 115 | 34 | 23 | 80 | 58 | 143 | 128 | 157 | 152 | 115 | 93 | 50 | 49 |
| **P (mg/30 g serving)** | 83 | 26 | 26 | 63 | 44 | 86 | 76 | 84 | 65 | 76 | 88 | 45 | 61 |
| **Al additive** | Na Al SO_4_ | Na Al SO_4_ | Na Al SO_4_ |  |  |  |  |  |  |  | Na Al SO_4_ | Na Al SO_4_ |  |
| **Al (mg/tortilla)** | 0.48 | 1.76 | 12.87 | 0.82 | 0.40 | 0.77 | 0.68 | 0.58 | 0.76 | 0.65 | 22.40 | 7.57 | 0.64 |
| **Al (mg/30 g serving)** | 0.35 | 1.33 | 14.83 | 0.65 | 0.31 | 0.47 | 0.41 | 0.31 | 0.33 | 0.43 | 21.04 | 6.77 | 0.80 |

^1^ Tortilla weight and element concentrations are averages of one or two tortillas (noted in the source).

Ag was below the MDL (0.0010) in all samples. As was ≤ 0.02 in all samples. Cd was ≤ 0.01 in corn and ≤ 0.03 in flour samples. Pb was ≤ 0.05 in all samples. U was ≤ 0.02 in all samples.V was ≤ 0.05 in all samples.

Outliers highlighted in red

**Table S8. Average P and Al concentration in flour tortillas**

|  | **Total Tortilla Weight (g) ^1^** | **P (mg/kg)** | **Al (mg/kg)** | **P (mg/tortilla)** | **P (mg/30 g serving)** | **Al (mg/tortilla)** | **Al (mg/30 g serving)** |
| --- | --- | --- | --- | --- | --- | --- | --- |
| average (mean) all results | 42 | 2111 | 123 | 92.1 | 63.3 | 3.9 | 3.7 |
| median all results | 39.8 | 2170 | 15.6 | 93.3 | 65.1 | 0.76 | 0.47 |
| average (mean) non-outliers | 42 | 2111 | 17.9 | 92.1 | 63.3 | 0.76 | 0.54 |
| median non-outliers | 39.8 | 2170 | 14 | 93.3 | 65.1 | 0.67 | 0.42 |
| are all data normally distributed? | yes | yes | no | yes | yes | no | no |
| are non-outliers normally distributed? | yes | yes | no | yes | yes | no | no |
