# Supplementary material for: The dietary burden of phosphorus and aluminum in ready-to-eat wheat flour tortillas exceeds that of corn tortillas: Implications for patients with chronic kidney disease
Source: Food Humanit. Author manuscript; Available in PMC 2026 Jul 23. (PMC13390896; doi:10.1016/j.foohum.2026.101060)
Supplement: Supplementary file 3 [file NIHMS2196054-supplement-Supplementary_file_3.docx]

**Table S9. Elemental concentrations measured per serving compared to listed concentrations**

| Corn Tortillas (Serving Size) | **Hard or Soft** | Na Content Packaging (mg) | Na Content Measured (mg) | Ca Content Packaging (mg) | Ca Content Measured (mg) | Fe Packaging (mg) | Fe Content Measured (mg) | K Packaging (mg) | K Content Measured (mg) | Reference |
| --- | --- | --- | --- | --- | --- | --- | --- | --- | --- | --- |
| Calidad (per 47 g serving) | **S** | **20** | **17** | 20 | 18 | 0.3 | 0.7 | 80 | 73 | Food4Less, Calidad White Corn Tortillas, 2024, URL: https://www.food4less.com/p/calidad-white-corn-tortillas/0007794800602 |
| Del Taco (2) | H |  |  |  |  |  |  |  |  |  |
| DelTaco (2) | S |  |  |  |  |  |  |  |  |  |
| El Comal (per 26 g serving) | S | 0 | 3 | 8 | 31 | 0 | 0.2 | 41 | 60 | EL COMAL, El Comal Corn Tortillas 70ct, Accessed 2024, URL: https://www.elcomal.com/products/corn-tortillas/el-comal-corn-tortillas-70ct/ |
| El Milagro (per 41 g serving) | S | 10 | 0.4 | 174 | 82 | 1 | 0.3 | 81 | 48 | Kroger, El Milagro Corn Tortillas, Accessed 2024, URL: https://www.kroger.com/p/el-milagro-corn-tortillas/0003862262117 |
| Guerrero (per 47 g serving) | S | 20 | 20 | 20 | 15 | 0.3 | 0.3 | 80 | 76 | Guerrero, White Corn Tortillas, Accessed 2024, URL: https://guerrerotortillas.com/products/white-corn-tortillas/ |
| Great Value (per 46 g serving) | S | 20 | 18 | 20 | 16 | 0.3 | 0.4 | 80 | 75 | Walmart, Great Value Medium White Corn Tortillas, Accessed 2024, URL: https://www.walmart.com/ip/Great-Value-Medium-White-Corn-Tortillas-30-Count/361076448 |
| La Banderita (per 47 g serving) | S | 15 | 18 | 18 | 14 | 0 | 0.3 | 52 | 74 | Olé, La Banderita Yellow Corn, Accessed 2024, URL: https://olemex.com/products/la-banderita-yellow-corn/ |
| La Pericos (2) | H |  |  |  |  |  |  |  |  |  |
| Mission (per 45 g serving) | S | 20 | 17 | 20 | 15 | 0.3 | 0.3 | 80 | 67 | MissionFoods, Street Tacos White Corn Tortillas, Accessed 2024, URL: https://www.missionfoods.com/products/street-tacos-white-corn-tortillas/ |
| Old El Paso (2) | H |  |  |  |  |  |  |  |  |  |
| Romero's (per 24 g serving) | S | 16 | 7 | 7 | 9 | 0 | 0.2 | 32 | 37 | nutritionix, Romero's Whole Grain Corn Tortillas, Accessed 2024, URL: https://www.nutritionix.com/i/romeros/whole-grain-corn-tortillas/62348edb5b63b0000847e4f8 |
| Taco Bell (1) | S |  |  |  |  |  |  |  |  |  |
| Taco Bell (1) | H |  |  |  |  |  |  |  |  |  |
|  |  |  |  |  |  |  |  |  |  |  |
| Flour Tortillas (Serving Size) |  |  |  |  |  |  |  |  |  |  |
| Calidad (per 41 g serving) | S | 370 | 369 | 100 | 101 | 1.4 | 1.6 | 40 | 40 | nutritionix, Calidad Flour Tortillas, Accessed 2024, URL: https://www.nutritionix.com/i/calidad-brand/flour-tortillas/61d3001337f1e100064f8987 |
| Diana’s (2) | S |  |  |  |  |  |  |  |  |  |
| Del Taco (2) | S |  |  |  |  |  |  |  |  |  |
| El Comal (per 43 g serving) | S | 110 | 272 | 19 | 60 | 1 | 0.3 | 34 | 57 | nutritionix, El Comal Flour Tortillas, Accessed 2024, URL: https://www.nutritionix.com/i/el-comal/flour-tortillas/5ac86d39be0112522908135e |
| Guerrero (per 58 g serving) | S | 370 | 307 | 100 | 50 | 1.5 | 1.6 | 40 | 51 | Guerrero, Caseras Soft Taco Flour Tortillas, Accessed 2024, URL: https://guerrerotortillas.com/products/caseras-soft-taco-flour-tortillas/ |
| Great Value (per 49 g serving) | S | 340 | 388 | 100 | 110 | 0.4 | 0.4 | 30 | 43 | Walmart, Great Value Flour Tortillas, Accessed 2024, URL: https://www.walmart.com/ip/Great-Value-Medium-Soft-Taco-Flour-Tortillas-17-5-oz-Bag-10-Count-Shelf-Stable/953466267 |
| Kroger (per 50 g serving) | S | 370 | 406 | 90 | 112 | 1.8 | 1.8 | 30 | 45 | Kroger, Kroger Soft Taco Size Flour Tortillas, Accessed 2024, URL: https://www.kroger.com/p/kroger-soft-taco-size-flour-tortillas/0001111005265 |
| La Banderita (per 45 g serving) | S | 220 | 325 | 56 | 67 | 1 | 2.2 | 35 | 39 | HEB, La Banderita Soft Taco Flour Tortillas, Accessed 2024, URL: https://www.heb.com/product-detail/la-banderita-soft-taco-flour-tortillas/2268511 |
| Mission (per 66 g serving) | S | 330 | 554 | 80 | 122 | 2 | 2.6 | 50 | 58 | Mission, Mission Flour Tortillas, Accessed 2024, URL: https://www.missionfoods.com/products/tortillas-caseras-flour-tortillas/ |
| Mas y Mas (per 45 g serving) | S | 410 | 362 | 100 | 103 | 1.5 | 1.5 |  |  | Kroger, Mas Y Mas Flour Tortillas, Accessed 2024, URL: https://www.kroger.com/p/mas-y-mas-soft-taco-size-flour-tortillas/0001111003843 |
| Old El Paso per 50 g serving) | S | 300 | 287 | 50 | 52 | 1.75 | 1.1 | 100 | 99 | Walmart, Old El Paso Soft Tortillas Medium, Accessed 2024, URL: https://www.walmart.ca/en/ip/Old-El-Paso-Soft-Tortillas-Medium/6000188866196 |
| Romero (per 39 g serving) | S | 234 | 230 | 60 | 69 | 0 | 1.1 | 29 | 51 | nutritionix,Romero's Flour Tortillas, Accessed 2024, URL: https://www.nutritionix.com/i/romeros/flour-tortillas-low-carb/51c53ed697c3e6efadd5aac9 |
| Taco Bell (per 60 g serving) | S | 380 | 521 | 40 | 124 | 2 | 2.4 |  |  | calorieking, Taco Bell Home Original Flour Tortillas, Accessed 2024, URL: https://www.calorieking.com/us/en/foods/f/calories-in-packaged-meals-flour-tortillas/X9gtnxAbTEi4ay-fsc-Alw |
