# Supplementary material for: The dietary burden of phosphorus and aluminum in ready-to-eat wheat flour tortillas exceeds that of corn tortillas: Implications for patients with chronic kidney disease
Source: Food Humanit. Author manuscript; Available in PMC 2026 Jul 23. (PMC13390896; doi:10.1016/j.foohum.2026.101060)
Supplement: Supplementary file 1 [file NIHMS2196054-supplement-Supplementary_file_1.docx]

**Figure S1.** Maize and wheat/flour tortilla elemental content other than phosphorus and aluminum, and results of statistical analysis. Maize tortillas had a statistically significantly greater concentration of magnesium, nickel, potassium, and zinc. Wheat/flour tortillas had a statistically significantly greater concentration of calcium, iron, manganese, selenium, sodium, and strontium.
